# First definitive record of Abelisauridae (Theropoda: Ceratosauria) from the Cretaceous Bahariya Formation, Bahariya Oasis, Western Desert of Egypt

**DOI:** 10.1098/rsos.220106

**Published:** 2022-06-08

**Authors:** Belal S. Salem, Matthew C. Lamanna, Patrick M. O'Connor, Gamal M. El-Qot, Fatma Shaker, Wael A. Thabet, Sanaa El-Sayed, Hesham M. Sallam

**Affiliations:** ^1^ Department of Geology, Faculty of Science, Benha University, Benha, Egypt; ^2^ Mansoura University Vertebrate Paleontology Center (MUVP), Department of Geology, Faculty of Science, Mansoura University, Mansoura, Egypt; ^3^ Department of Biological Sciences, Ohio University, 228 Irvine Hall, Athens, OH, USA; ^4^ Ohio Center for Ecological and Evolutionary Studies, Ohio University, Athens, OH, USA; ^5^ Section of Vertebrate Paleontology, Carnegie Museum of Natural History, Pittsburgh, PA, USA; ^6^ Department of Biomedical Sciences, Ohio University Heritage College of Osteopathic Medicine, Ohio University, Athens, OH, USA; ^7^ Egyptian Environmental Affairs Agency, Cairo, Egypt; ^8^ Department of Earth and Environmental Sciences, University of Michigan, Ann Arbor, MI, USA; ^9^ Institute of Global Health and Human Ecology (I-GHHE), School of Sciences and Engineering, American University in Cairo, New Cairo, Egypt

**Keywords:** Abelisauridae, Egypt, Africa, Cretaceous, Bahariya Formation

## Abstract

Numerous non-avian theropod dinosaur fossils have been reported from the Upper Cretaceous (Cenomanian) Bahariya Formation, Bahariya Oasis, Western Desert of Egypt, but unambiguous materials of Abelisauridae have yet to be documented. Here we report Mansoura University Vertebrate Paleontology Center (MUVP) specimen 477, an isolated, well-preserved tenth cervical vertebra of a medium-sized abelisaurid from the Bahariya Formation. The new vertebra shows affinities with those of other Upper Cretaceous abelisaurids from Madagascar and South America, such as *Majungasaurus crenatissimus*, *Carnotaurus sastrei*, *Viavenator exxoni* and a generically indeterminate Patagonian specimen (Museo Padre Molina specimen 99). Phylogenetic analysis recovers the Bahariya form within Abelisauridae, either in a polytomy of all included abelisaurids (strict consensus tree) or as an early branching member of the otherwise South American clade Brachyrostra (50% majority rule consensus tree). MUVP 477, therefore, represents the first confirmed abelisaurid fossil from the Bahariya Formation and the oldest definitive record of the clade from Egypt and northeastern Africa more generally. The new vertebra demonstrates the wide geographical distribution of Abelisauridae across North Africa during the middle Cretaceous and augments the already extraordinarily diverse large-bodied theropod assemblage of the Bahariya Formation, a record that also includes representatives of Spinosauridae, Carcharodontosauridae and Bahariasauridae.

## 1. Introduction

Abelisaurid ceratosaurs were among the most diverse and geographically widespread medium- to large-bodied theropod dinosaurs during the Cretaceous in the Eurogondwanan landmasses, occupying carnivorous niches in South America, continental Africa, Indo-Madagascar, Europe and possibly Australia [1–4]. Nevertheless, despite the rich and ever-increasing middle and Late Cretaceous non-avian dinosaur record of Egypt (e.g. [5–8]), only highly fragmentary evidence of Abelisauridae has as yet come to light from this nation and northeastern Africa in general. At present, the only unquestioned abelisaurid fossil from Egypt is an isolated tooth from an exposure of the uppermost Cretaceous (Campanian–Maastrichtian) Duwi Formation near Idfu in the southern Nile Valley region [9,10]. Moreover, a few additional, possible abelisaurid (or abelisauroid) skeletal elements have been reported from the same general geographical area: two ungual phalanges from the Duwi Formation [9] and the proximal end of a small tibia from the slightly older (Campanian) Nubian Sandstone [2,11]. Other putative abelisauroid materials have been recovered from the Western Desert of Egypt, and include the proximal portion of a left fibula from the Campanian Quseir Formation of the Kharga Oasis [8] and tooth crowns [12] and appendicular bones from the Cenomanian Bahariya Formation of the Bahariya Oasis; some of the latter were referred to cf. *Elaphrosaurus bambergi* or aff. *Erectopus sauvagei* by Stromer [5,13]; see also Rauhut & Werner [14], who regarded the former taxon as a ‘probable ceratosaur’.

Here we broaden the distribution of Abelisauridae by describing the first definitive fossil of this theropod group from the Bahariya Formation, an isolated but well-preserved cervical vertebra identified as the tenth in the series. The discovery increases dinosaur diversity in the Bahariya Formation by expanding the list of non-avian theropod taxa documented from this geologic unit, which currently includes the spinosaurid *Spinosaurus aegyptiacus* [15], the carcharodontosaurid *Carcharodontosaurus saharicus* [16], the bahariasaurid *Bahariasaurus ingens* [13] (and possibly the bahariasaurid *Deltadromeus agilis* [17], if it is distinct from *B*. *ingens*; see [2]) and indeterminate forms [5,12,13]. Intriguingly, it adds yet another taxon to the already exceptionally speciose large-bodied theropod fauna of this formation and further underscores the similarity of the Bahariya dinosaur assemblage to that of the penecontemporaneous Kem Kem Group of northwestern Africa [17–20].

Institutional abbreviations—MACN-CH, Museo Argentino de Ciencias Naturales, Colección Chubut, Buenos Aires, Argentina; MAU, Museo Argentino Urquiza, Rincón de los Sauces, Argentina; MPM, Museo Padre Molina, Río Gallegos, Argentina; MUVP, Mansoura University Vertebrate Paleontology Center, Mansoura, Egypt; UA, Université d’Antananarivo, Antananarivo, Madagascar.

## 2. Material and methods

A 2016 expedition to the Bahariya Oasis by an MUVP team discovered multiple fossil remains from the Bahariya Formation, including the vertebra described in this work. The specimen was surface collected from near Gebel El Dist in the northern part of the Bahariya Oasis depression. The vertebra was originally covered in many places by an iron-rich concretionary matrix. It has since been fully mechanically prepared, reconstructed and reposited in the permanent collection of the MUVP in Mansoura, Egypt, under catalogue number MUVP 477. Nomenclature of vertebral neural arch laminae herein follows Wilson [21]; that of fossae follows Wilson *et al*. [22].

### 2.1. Systematic palaeontology

Theropoda [23].

Ceratosauria [24].

Abelisauroidea [1].

Abelisauridae [25].

Referred specimen—MUVP 477, an isolated caudal (tenth) cervical vertebra.

**Figure 1.**
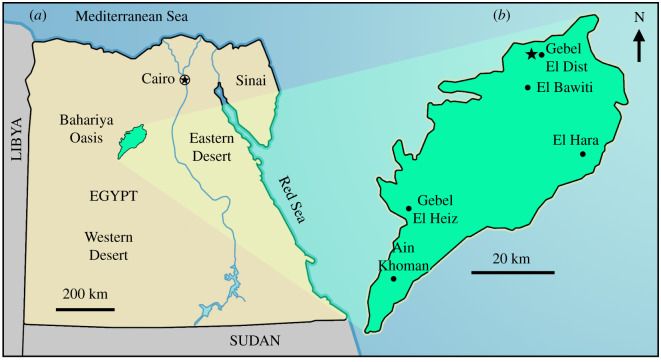
Location of the fossil locality. (*a*) Map of Egypt showing location of the Bahariya Oasis, Western Desert of Egypt (green). (*b*) Site (indicated by star) that produced the abelisaurid vertebra (MUVP 477) described herein, in the Gebel El Dist region of the Bahariya Oasis.

Locality and horizon—Gebel El Dist region, northern area of Bahariya Oasis depression (figure 1), Western Desert of Egypt. Upper Cretaceous (Cenomanian) Bahariya Formation.

Taxonomic comments—MUVP 477 exhibits features consistent with assignment to Abelisauroidea. These include: (1) dorsal surface of neural arch clearly delimited from lateral surface of diapophysis; (2) deep spinopre- and spinopostzygapophyseal fossae; and (3) well-developed epipophyses. Furthermore, according to the results of our phylogenetic analysis, the dorsoventrally tall, anteroposteriorly short neural arch is an unambiguous synapomorphy of Abelisauridae (within Abelisauroidea), permitting the referral of the new vertebra to the former, less inclusive clade.

### 2.2. Description

**Figure 2.**
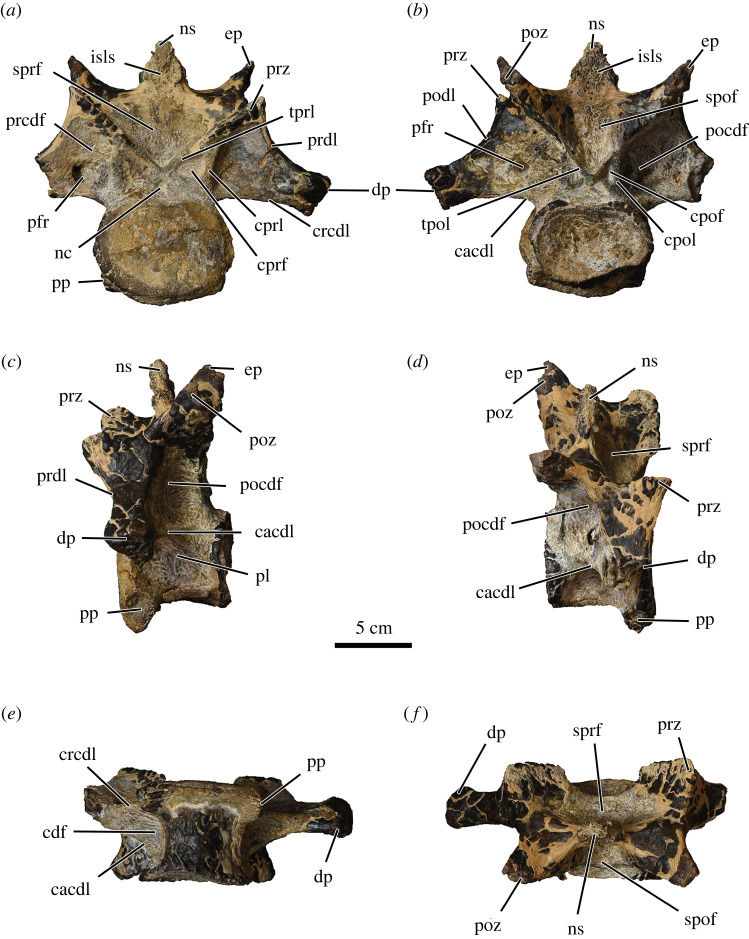
Tenth cervical vertebra of Abelisauridae indet. (MUVP 477) in cranial (*a*), caudal (*b*), left lateral (*c*), right dorsolateral (*d*), ventral (*e*) and dorsal (*f*) views. cacdl, caudal centrodiapophyseal lamina; cdf, centrodiapophyseal fossa; cpof, centropostzygapophyseal fossa; cpol, centropostzygapophyseal lamina; cprf, centroprezygapophyseal fossa; cprl, centroprezygapophyseal lamina; crcdl, cranial centrodiapophyseal lamina; dp, diapophysis; ep, epipophysis; isls, interspinous ligament scar; nc, neural canal; ns, neural spine; pfr, pneumatic foramen; pl, pleurocoel; pocdf, postzygapophyseal centrodiapophyseal fossa; podl, postzygodiapophyseal lamina; poz, postzygapophysis; pp, parapophysis; prcdf, prezygapophyseal centrodiapophyseal fossa; prdl, prezygodiapophyseal lamina; prz, prezygapophysis; spof, spinopostzygapophyseal fossa; sprf, spinoprezygapophyseal fossa; tpol, intrapostzygapophyseal lamina; tprl, intraprezygapophyseal lamina.

MUVP 477 is the tenth cervical vertebra (C10) of an abelisaurid theropod dinosaur (figure 2; table 1). It is larger than C10 of *Majungasaurus crenatissimus* [27] and the putative first dorsal vertebra (D?1) of *Dahalokely tokana* [29], but smaller than C10 of *Carnotaurus sastrei* [26]. It is well preserved and nearly complete, lacking only the lateral part of the right diapophysis and the ventral margin of the caudal articular surface.

**Table 1.** Measurements (mm) of tenth cervical vertebra (C10) of Abelisauridae indet. (MUVP 477), *Carnotaurus sastrei* (MACN-CH 894 [26]), *Majungasaurus crenatissimus* (UA 8678 [27]), *Viavenator exxoni* (MAU-Pv-LI-530 [28]) and *Dahalokely tokana*, D?1 (UA 9855 [29]). Table modified from O'Connor [27]. CENL, centrum length (= maximum craniocaudal length of centrum); CDCW, caudal centrum width (= maximum transverse width of caudal articular facet of centrum); CDCH, caudal centrum height (= maximum dorsoventral height of caudal articular facet of centrum); MIDW, midcentral width (= transverse width at centrum midlength); TOVH, total vertebral height (= total dorsoventral extent of vertebra including centrum and neural spine); NSH, neural spine height (= dorsoventral extent of neural spine measured from dorsal margin of neural canal); NSL, neural spine length (= craniocaudal extent of neural spine at spine midheight); NSW, neural spine width (= transverse extent of neural spine at spine midheight); IZW, interzygapophyseal width (= distance between lateral margins of postzygapophyses); IZL, interzygapophyseal length (= distance from cranial margin of right prezygapophysis to caudal margin of right postzygapophysis); IPPW, interparapophyseal width (= distance between lateral limits of parapophyses); IDPW, interdiapophyseal width (= distance between lateral limits of diapophyses); PP/DP, para-diapophyseal index (= ratio of interparapophyseal width to interdiapophyseal width); EPL, epipophyseal length (= distance from caudal margin of postzygapophyseal facet to caudalmost extent of epipophysis). *Incomplete measurement due to missing bone (e.g. partial breakage of a transverse process). †Unable to measure due to damaged/missing bone. —, Measurement not reported for element.

	CENL	CDCW	CDCH	MIDW	TOVH	NSH	NSL	NSW	IZW	IZL	IPPW	IDPW	PP/DP	EPL
MUVP 477	67.00	79.00	61.00	55.00	170.50	95.00	52.00	13.80	94.00	79.00	84.41	195.00*	0.43	9.17
*Dahalokely* (UA 9855) (D?1)	44.00	48.50	39.80	30.60	108.60	57.20	9.60	17.50	57.80	54.50	64.50	†	†	—
*Carnotaurus* (MACN-CH 894)	94.00	112.00	100.00	82.00	242.00	39.00	31.00	36.00	—	—	—	—	—	51.00
*Majungasaurus* (UA 8678)	58.40	56.80	62.10	36.80*	136.40	63.60	11.70	18.70	86.90	70.50	54.20*	134.30*	0.40	2.10
*Viavenator* (MAU-Pv-LI-530)	72.00	55.90	55.00	—	184.00	43.20	—	—	—	—	79.20	207.00	0.38	19.80

**Figure 3.**
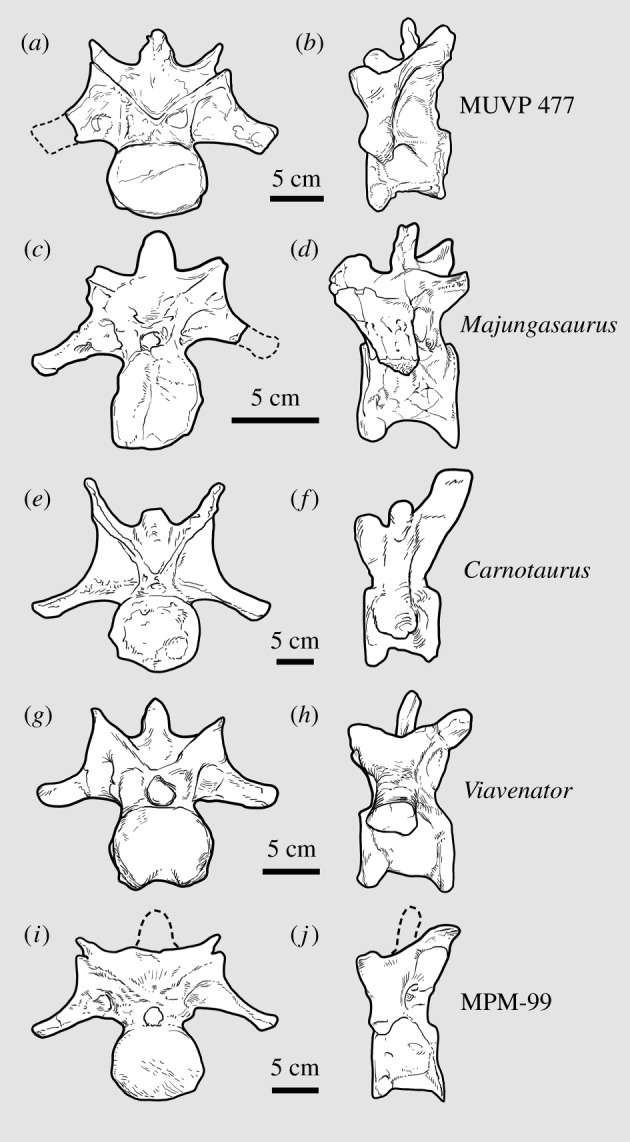
Tenth cervical vertebra of representative abelisaurids in cranial (*a*,*c*,*e*,*g*,*i*) and left lateral (*b*,*d*,*f*,*h*,*j*) views. (*a*,*b*) Abelisauridae indet. (MUVP 477) from Egypt. (*c*,*d*) *Majungasaurus crenatissimus* (UA 8678) after [27]. (*e*,*f*) *Carnotaurus sastrei* (MACN-CH 894) after [26]. (*g*,*h*) *Viavenator exxoni* (MAU-Pv-LI-530) after [28,30]. (*i*,*j*) Abelisauridae indet. from Argentina (MPM-99) after [31]. Cranial views (*a*,*c*,*e*,*g*,*i*) scaled to same transverse width to facilitate comparisons. Dashed lines in (*a*,*c*,*i*,*j*) represent reconstructed areas.

The centrum is craniocaudally shorter than transversely wide, as in many other abelisaurids (figure 3; table 1) but contrasting strongly with the much more elongate caudal cervical centra of many non-abelisaurid ceratosaurs such as *Berthasaura leopoldinae* [32], *Elaphrosaurus bambergi* [33–35], *Limusaurus inextricabilis* [36], *Masiakasaurus knopfleri* [37,38] and *Vespersaurus paranaensis* [39]. The centrum lacks a ventral keel, as is also the case in *Majungasaurus* [27] and *Viavenator exxoni* [28,30]. In ventral view, a marked constriction is observed in the middle part of the centrum, as in caudal cervical vertebrae of many taxa within Abelisauria (= Noasauridae + Abelisauridae) [3,40,41] such as *Carnotaurus*, *Majungasaurus*, *Viavenator*, the generically indeterminate Patagonian mid-Cretaceous abelisaurid MPM-99 [31] and (less markedly) *Ekrixinatosaurus novasi* [42], *Elaphrosaurus* [34,35] and *Masiakasaurus* [37].

The centrum possesses a flat to slightly concave cranial articular facet, as in *Viavenator* and *Majungasaurus*, and a strongly concave caudal facet as in the caudalmost cervical vertebrae of *Eoabelisaurus mefi* [43]. The cranial and caudal articular surfaces are oval, being transversely wider than dorsoventrally high, similar to the condition in *Eoabelisaurus* in which the middle and caudal cervical centra are notably wider than high. These surfaces have a concave dorsal border, whereas the ventral border of the cranial articular surface is rounded; the ventral border of the caudal articular surface is not preserved.

The centrum exhibits a single pleurocoel on each lateral surface, developed as a deep depression caudodorsal to the parapophysis. In *Majungasaurus*, by contrast, the centrum of C10 has a distinct pneumatic foramen at the cortical surface (albeit one with multiple foramina set within it) immediately caudodorsal to the parapophysis (see [27, fig. 9d]), whereas cervical vertebrae of South American abelisaurids (e.g. *Carnotaurus*, *Ekrixinatosaurus*, *Viavenator*, MPM-99) exhibit two distinct pleurocoels on each lateral surface, one positioned caudodorsal to the parapophysis and the other near the caudal border of the centrum. The parapophyses of MUVP 477 are situated at the cranioventral edge of the centrum and are lateroventrally projected. The parapophyseal facets are slightly ventrally inclined and oval, with the long axis oriented dorsoventrally. The centrum has camellate interior pneumatic structure as in other abelisaurids and some non-abelisaurid ceratosaurs.

The diameter of the neural canal is small, as in *Carnotaurus*, in contrast to the larger neural canal of *Viavenator* (figure 3). The neural arch is dorsoventrally tall and anteroposteriorly short, a condition that optimizes as an unambiguous synapomorphy of Abelisauridae in our phylogenetic analysis (see below). The diapophyses (i.e. transverse processes) are well developed, subtriangular in cross-section and ventrolaterally directed, with the long axis of each forming an angle of approximately 65° relative to the midsagittal plane (i.e. the dorsoventral axis of the vertebra), as in *Majungasaurus* [27]. The diapophyses merge with the centrum via the cranial (= anterior) [21] and caudal (= posterior) centrodiapophyseal laminae and with the neural arch via the pre- and postzygodiapophyseal laminae. The diapophyses are dorsally located, as in *Majungasaurus* [27], *Dahalokely* [29] and MPM-99, whereas in *Carnotaurus* [26] and *Viavenator* [28,30] the diapophyses largely obscure the parapophyses in lateral view.

Centroprezygapophyseal fossae are present on the cranial surface of the neural arch, but there is only very limited expression of the centropostzygapophyseal fossae on the caudal surface; thus, the centropostzygapophyseal fossae are not as developed in MUVP 477 as they are in *Carnotaurus*, *Ekrixinatosaurus*, *Majungasaurus* and MPM-99 [26]. The prezygapophyseal centrodiapophyseal fossae are bounded by the centroprezygapophyseal, prezygodiapophyseal and cranial centrodiapophyseal laminae, and each fossa is excavated by a deep, possibly pneumatic foramen. The postzygapophyseal centrodiapophyseal fossae are bordered by the centropostzygapophyseal, postzygodiapophyseal and caudal centrodiapophyseal laminae. The left postzygapophyseal centrodiapophyseal fossa is pierced by another foramen that may also be pneumatic in nature. Conversely, and interestingly, the centrodiapophyseal fossae are imperforate, a morphology that optimizes as a unique reversal of a neoceratosaurian synapomorphy.

The prezygapophyses are strongly dorsomedially inclined, with large articular facets that are subtriangular in dorsal view with their long axis oriented mediolaterally, similar in shape to those of C10 in other undoubted abelisaurids and D?1 of *Dahalokely* [29]. They are cranially projected but only slightly extend past the cranial border of the centrum, as in *Viavenator* [28], *Carnotaurus* [26] and MPM-99 [31], whereas this projection is more pronounced in *Majungasaurus*. The postzygapophyses are steeply ventrolaterally inclined, but detailed morphology of the articular facets is difficult to determine due to a dark brown coating of permineralization. Relatively small but well-developed epipophyses emerge dorsal to the postzygapophyses and are laterally and caudally projected. The conformation of the epipophyses is most similar to those observed in *Majungasaurus*, MPM-99 and *Viavenator* (figure 3); the epipophyses of *Carnotaurus*, by contrast, are hypertrophied through the caudalmost cervical vertebrae [26].

The neural spine is craniocaudally compressed and transversely wide, as in *Carnotaurus*, *Ekrixinatosaurus* and possibly *Rahiolisaurus gujaratensis*. It is located dorsal to the caudal half of the centrum, and is taller than the epipophyses, as in *Majungasaurus* and *Viavenator* (figure 3). In *Carnotaurus*, conversely, the epipophyses are taller than the neural spine [26]. The spinopre- and spinopostzygapophyseal fossae are deep, but the latter are shallower and less extensive than the deeper, wider spinoprezygapophyseal fossae. A rough, irregular zone on both the cranial and caudal surfaces of the neural spine, also observed in *Viavenator* [28], *Carnotaurus* and *Majungasaurus*, is interpreted as an insertion area for interspinous ligament.

### 2.3. Estimated body size

We estimated the total body length of the abelisaurid individual represented by MUVP 477 by applying the method of Grillo & Delcourt [44] to the dimensions of the new vertebra (table 1). We calculated body length estimates of 5.30 m based on centrum length, 6.31 m based on centrum width and 5.79 m based on centrum height (average = 5.77 m). These estimated lengths indicate that the individual represented by MUVP 477 was medium-sized relative to other members of Abelisauridae. Other abelisaurid taxa with comparable estimated body lengths (i.e. 4–7 m) include *Majungasaurus*, *Viavenator* and *Xenotarsosaurus bonapartei* [45].

### 2.4. Phylogenetic analysis

**Figure 4.**
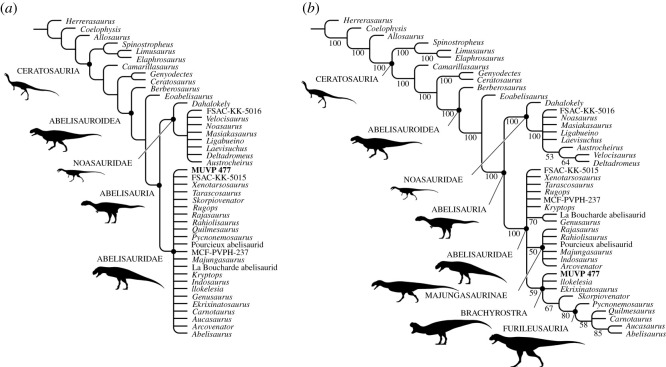
Phylogenetic position of Abelisauridae indet. (MUVP 477). Strict (*a*) and 50% majority rule (*b*) consensus trees of 1640 most parsimonious trees (MPTs) of 758 steps recovered by parsimony analysis (Tree analysis using New Technology v. 1.1 [46]) of the data matrix presented by Smyth *et al*. [41] with the addition of MUVP 477. Node names mostly follow Tortosa *et al*. [3] and Smyth *et al*. [41]; numerals in (*b*) are percentages of MPTs. Note that several recent works have regarded *Camarillasaurus cirugedae* and *Deltadromeus agilis* as members of Tetanurae rather than Ceratosauria; specifically, Samathi *et al*. [47] reappraised *Camarillasaurus* as a representative of Spinosauridae, and Apesteguía *et al*. [48] and Motta *et al*. [49] regarded *Deltadromeus* as a member of Bahariasauridae, a clade with potential affinities to Megaraptora. As such, we regard the phylogenetic positions of these two forms depicted above with caution. All ceratosaur silhouettes from PhyloPic (http://phylopic.org/); individual credits as follows: Ceratosauria (FunkMonk); Abelisauroidea (Iain Reid); Noasauridae, Majungasaurinae and Furileusauria (Scott Hartman); Abelisauria (Andrew Farke and Joseph Sertich); Abelisauridae and Brachyrostra (Jagged Fang Designs).

We conducted a phylogenetic analysis using the data matrix of Smyth *et al*. [41], with the addition of MUVP 477 (figure 4). We analysed the matrix using Tree analysis using New Technology (TNT) v. 1.1 [46] under equally weighted maximum parsimony. A heuristic search (1000 replications of Wagner trees, random seed 1, random addition sequence, tree bisection-reconnection branch swapping algorithm, ten trees held per replication, zero-length branches collapsed) recovered 1640 most parsimonious trees (MPTs) of 758 steps (Consistency Index = 0.579; Retention Index = 0.709). MUVP 477 was positioned within Abelisauridae, either in a polytomy of all included abelisaurids (strict consensus tree; figure 4*a*) or, more interestingly, as an early branching member of the otherwise South American clade Brachyrostra (50% majority rule consensus tree; figure 4*b*). Nevertheless, we regard the potential identity of MUVP 477 as a brachyrostran abelisaurid with caution given that (1) Brachyrostra is recovered in only 59% of the MPTs (figure 4*b*); (2) this clade has not previously been documented from Afro-Arabia; and (3) the specimen consists of only a single cervical vertebra.

## 3. Discussion

The isolated, well-preserved tenth cervical vertebra (MUVP 477) reported herein constitutes the first indisputable evidence of Abelisauridae from the Upper Cretaceous (Cenomanian) Bahariya Formation of the Bahariya Oasis of the Western Desert of Egypt. The specimen indicates the presence of a medium-sized abelisaurid with affinities to Upper Cretaceous taxa from Madagascar and South America such as *Majungasaurus*, *Carnotaurus*, *Viavenator* and the generically indeterminate form MPM-99. The results of our phylogenetic analysis and the presence of several widely recognized synapomorphies of Abelisauroidea (dorsal surface of neural arch clearly delimited from lateral surface of diapophysis, deep spinopre- and spinopostzygapophyseal fossae, well-developed epipophyses) permit the assignment of MUVP 477 to this clade, with another synapomorphic character state (dorsoventrally tall, anteroposteriorly short neural arch) justifying its referral to the less inclusive Abelisauridae.

Prior to the discovery of MUVP 477, abelisaurids were definitively represented in Egypt, and northeastern Africa more generally, only by an isolated tooth from the uppermost Cretaceous (Campanian–Maastrichtian) Duwi Formation of the southern Nile Valley [9,10]. The new vertebra, therefore, extends the stratigraphic record of Abelisauridae from northeastern Africa into the earliest part of the Late Cretaceous (Cenomanian).

**Figure 5.**
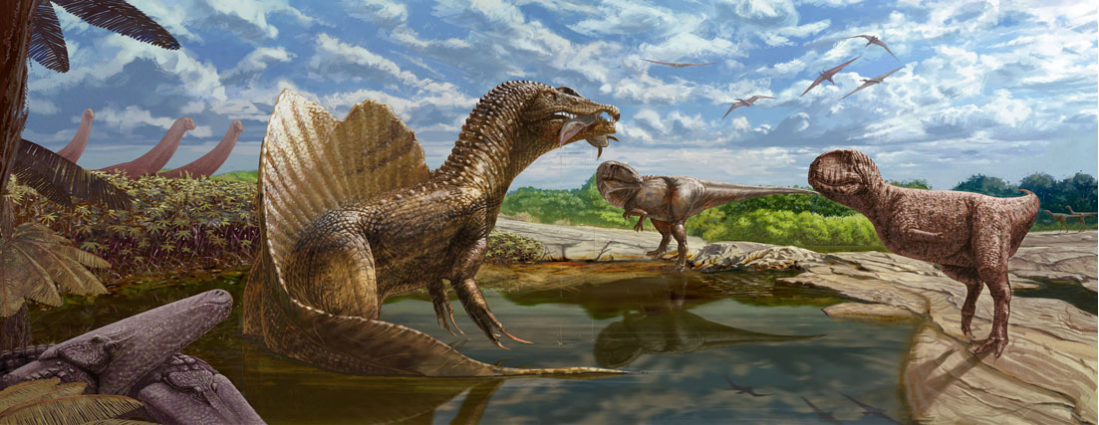
Reconstruction of the palaeoecosystem of the Upper Cretaceous (Cenomanian) Bahariya Formation of the Bahariya Oasis, Western Desert of Egypt, showing diversity of large-bodied theropod dinosaurs. In the foreground, the unidentified abelisaurid described herein (right) confronts the spinosaurid *Spinosaurus aegyptiacus* (left centre, with dipnoan (lungfish) *Retodus tuberculatus* in jaws) and the carcharodontosaurid *Carcharodontosaurus saharicus* (right centre) while two individuals of the stomatosuchid crocodyliform *Stomatosuchus inermis* (left) look on. In the background, a herd of the titanosaurian sauropod *Paralititan stromeri* (left) warily regards these theropods and two individuals of the bahariasaurid *Bahariasaurus ingens* (far right) while a small flock of an undescribed pterosaur soars above. The vegetation is dominated by the mangrove-like tree fern *Weichselia reticulata*. Artwork by Andrew McAfee, Carnegie Museum of Natural History.

Multiple non-avian theropod dinosaur taxa have been reported from the Bahariya Formation, including the spinosaurid *Spinosaurus aegyptiacus* [13,15], the carcharodontosaurid *Carcharodontosaurus saharicus* [16], the bahariasaurid *Bahariasaurus ingens* [13] (and possibly the bahariasaurid *Deltadromeus agilis*, if it is distinct from *B*. *ingens* as argued by [17], but see [2]) and indeterminate forms, some of which were referred to cf. *Elaphrosaurus bambergi* or aff. *Erectopus sauvagei* by Stromer [5,13] and may have affinities with Ceratosauria and clades therein. Intriguingly, several of these forms—namely *Spinosaurus*, *Carcharodontosaurus* and *Bahariasaurus*/*Deltadromeus*—attained exceptionally large body sizes among non-avian theropod dinosaurs, comparable to that of *Tyrannosaurus rex* [6,20]. Though it undoubtedly pertains to a substantially smaller-bodied animal, the new abelisaurid vertebra (MUVP 477) confirms the presence of a fourth medium-sized to large (approximately 6 m in total body length) theropod taxon in the Bahariya Formation palaeoecosystem (figure 5).

The addition of an abelisaurid to the Bahariya Formation palaeofauna further increases the similarity of its theropod assemblage to that of the penecontemporaneous Kem Kem Group of eastern Morocco, from which these ceratosaurs have already been documented (e.g. [2,19,20,50,51]). In addition to abelisaurids, both units have also yielded spinosaurids (*Spinosaurus* and, in the case of the Kem Kem Group, perhaps one or more additional taxa, e.g. *Sigilmassasaurus brevicollis* [50,52,53]; but see [54]), carcharodontosaurids (*Carcharodontosaurus* and, again in the Kem Kem Group, potentially other forms, e.g. *Sauroniops pachytholus* [55]; but see [20]) and bahariasaurids (*Bahariasaurus*/*Deltadromeus*). Except for Bahariasauridae, all these theropod clades are also known from the Lower Cretaceous (Aptian–Albian) Elrhaz Formation of Niger, indicating that the abelisaurid/spinosaurid/carcharodontosaurid triumvirate of large carnivorous dinosaurs with distinctive skull architecture became established in northern Africa millions of years prior to the deposition of the Bahariya Formation and the Kem Kem Group [20,56,57]. Small-bodied noasaurid ceratosaurs [41] and dromaeosaurid paravians [20,58] have also been reported from the Kem Kem Group, and one or both of these clades may also be represented in the Bahariya Formation by isolated, as-yet-undescribed teeth [12] (M.C.L. 2022, personal observation).

The seemingly unusual abundance of carnivorous dinosaurs—especially exceptionally large-bodied taxa—and the simultaneous scarcity of their herbivorous counterparts in the Bahariya Formation were first noted by Stromer [5], and more recently dubbed ‘Stromer's Riddle’ [20,59–62]. This distinctive pattern has since been found to characterize other North African mid-Cretaceous continental palaeoecosystems (e.g. [20,57,63]), and, in particular, the Kem Kem Group [17,20,62,64]. Indeed, the discovery of the medium-sized Bahariya Formation abelisaurid represented by MUVP 477 further underscores the likelihood that a cosmopolitan large-bodied theropod fauna existed across northern Africa during the Cenomanian, from what is now the outcrop area of the Kem Kem Group in the west to that of the Bahariya Formation in the east, and potentially beyond. Interestingly, this palaeobiogeographic hypothesis stands in contrast to that proposed by Mannion & Barrett [65] on the basis of coeval sauropod occurrences; namely, that the emplacement of the Trans-Saharan Seaway during the mid-Cretaceous led to the divergence of Cenomanian terrestrial vertebrate faunas in northwestern and northeastern Africa.

Finally, in our view, the Bahariya Formation of the Bahariya Oasis holds considerable untapped potential to better characterize the still-enigmatic non-avian theropods—and indeed, other continental vertebrates—that inhabited northern Africa during the early stages of the Late Cretaceous. This is in part because, when compared with the more intensively sampled Kem Kem Group, the Bahariya Formation appears to exhibit a considerably greater propensity for yielding associated (sometimes partly articulated), phylogenetically informative partial skeletons of predominantly land-living vertebrate taxa. For example, palaeontological field efforts in the oasis during the early 20th and 21st centuries have yielded at least 17 associated partial skeletons of large-bodied non-avian dinosaurs, 11 of which have been described in detail to date [5,6,13,15,16,66,67] (B.S.S. 2018 and 2021, M.C.L. 2000 and 2001, H.M.S. 2018 and 2021, personal observations), whereas the Kem Kem Group has yielded only three well-documented associated dinosaur skeletons in total (the holotypes of *Deltadromeus* and the rebbachisaurid sauropod *Rebbachisaurus garasbae* plus a more recently discovered partial skeleton of *Spinosaurus*; see [20,64]). Although preservational assessments of Kem Kem Group dinosaurs are complicated by the widespread commercial trade in Moroccan fossils—for example, additional skeletons have almost certainly been discovered but not formally scientifically described (e.g. [50, p. 355])—the substantially greater number of associated skeletons from the Bahariya Formation (despite much less collecting effort having been expended in that formation, especially when the commercial trade is considered) suggests the existence of taphonomic distinctions between these two North African Cenomanian sedimentary units. If so, then these distinctions may be directly linked to palaeoenvironmental differences between these units. In particular, the predominant dinosaur-bearing horizons of the Bahariya Formation appear to represent a comparatively low-energy paralic habitat dominated by the mangrove tree fern *Weichselia reticulata* [6,68] (figure 5), whereas those of the Kem Kem Group (particularly the Gara Sbaa Formation) were apparently deposited in a substantially higher-energy fluvial system in which the complete disarticulation and dissociation of skeletal elements prior to and during entombment was common [20]. Further taphonomic studies of continental vertebrates from the Bahariya Formation and the Kem Kem Group are needed to evaluate these hypotheses.

## 4. Conclusion

The cervical vertebra (MUVP 477) described herein represents the first confirmed fossil of Abelisauridae from the Bahariya Formation, establishing it as the oldest definitive record of this theropod clade from Egypt and northeastern Africa more generally. The new vertebra demonstrates the wide geographical distribution of abelisaurids across North Africa during the middle Cretaceous and augments the already extraordinarily diverse large-bodied non-avian theropod record of the Bahariya Formation, a unit that also preserves representatives of Spinosauridae, Carcharodontosauridae and Bahariasauridae. This abelisaurid/spinosaurid/carcharodontosaurid/bahariasaurid faunal assemblage appears to have extended across most or all of northern Africa during the Cenomanian, suggesting that the Trans-Saharan Seaway did not represent a significant barrier to large-bodied theropod dispersal at this time. The Bahariya Formation holds unrealized potential to improve understanding of this northern African Cenomanian fauna due to the relative commonality of phylogenetically informative associated partial skeletons in this stratigraphic unit.

## Data Availability

The specimen is catalogued and accessible in the collections of the Mansoura University Vertebrate Paleontology Center (MUVP), Department of Geology, Faculty of Science, Mansoura University, Mansoura, Egypt. The phylogenetic data matrix is provided as electronic supplementary material [69].
